# Release from belowground enemies and shifts in root traits as interrelated drivers of alien plant invasion success: a hypothesis

**DOI:** 10.1002/ece3.1725

**Published:** 2015-09-25

**Authors:** Wayne Dawson

**Affiliations:** ^1^ Department of Biology, EcologyUniversity of KonstanzKonstanzGermany

**Keywords:** Competition, exotic plant, plant–soil feedback, post‐introduction evolution, soil microbiota

## Abstract

Our understanding of the interrelated mechanisms driving plant invasions, such as the interplay between enemy release and resource‐acquisition traits, is biased by an aboveground perspective. To address this bias, I hypothesize that plant release from belowground enemies (especially fungal pathogens) will give invasive plant species a fitness advantage in the alien range, *via* shifts in root traits (e.g., increased specific root length and branching intensity) that increase resource uptake and competitive ability compared to native species in the alien range, and compared to plants of the invader in its native range. Such root‐trait changes could be ecological or evolutionary in nature. I explain how shifts in root traits could occur as a consequence of enemy release and contribute to invasion success of alien plants, and how they could be interrelated with other potential belowground drivers of invasion success (allelopathy, mutualist enhancement). Finally, I outline the approaches that could be taken to test whether belowground enemy release results in increased competitive ability and nutrient uptake by invasive alien plants, via changes in root traits in the alien range.

## Introduction: Plant Traits, Invasions, and Enemy Release

Much of the study on alien plant invasions has focused on the central question “Are invaders different?” (Elton 1958; Baker 1965; Rejmanek and Richardson 1996; Pyšek and Richardson 2007; van Kleunen et al. 2010a,b). Most frequently, invasion ecologists have tried to answer this question by comparing either traits or biotic interactions involving invasive species to those of native or noninvasive aliens (van Kleunen et al. 2010a,b). Differences in either traits or biotic interactions would then indicate a role for the trait or interactions in driving invasion success. Invasive plant species are often considered to be fast‐growing (Grotkopp et al. 2002; Grotkopp and Rejmanek 2007; Dawson et al. 2011), to have higher fitness‐related traits (van Kleunen et al. 2010a,b), and to respond more positively to increases in resource availability (Davidson et al.*,* 2011; Palacio‐Lopez and Gianoli, 2011; Dawson et al. 2012a,b; Parepa et al. 2013; Seabloom et al. 2015), than native or noninvasive counterparts. These differences in traits and resource acquisition are thought to represent a competitive advantage for the invaders.

However, most trait‐based comparisons in invasion ecology have focused on easier‐to‐measure aboveground traits. For example, specific leaf area (SLA) is considered a component of the leaf economics spectrum (Wright et al. 2004), and of plant relative growth rate. Plants with greater SLA are considered to be faster‐growing, more competitive, resource‐acquisition species, which may characterize invaders. Specific root length (SRL) is often considered as a belowground analog to SLA (Reich 2014), representing the total length of root deployed per unit dry mass of root tissue invested and thus ability to acquire soil resources (nutrients and water). But, compared to traits related to light‐capturing ability of invasive plants, we know very little about the root traits determining their ability to acquire soil resources (Reich 2014).

For biotic interactions, considerable attention has been paid to the hypothesis that some alien plants benefit in the alien range due to leaving behind natural enemies (particularly specialists) from the native range, resulting in increased fitness and competitive ability compared to natives (the “Enemy Release” hypothesis—ERH; Keane and Crawley 2002). An extension of the ERH is that the absence of specialist enemies leads to selection against genotypes investing more in costly defenses against missing enemies, and selection in favor of genotypes investing more in growth, leading to greater competitive ability (the “Evolution of Increased Competitive Ability” hypothesis—EICA; Blossey and Notzold 1995). Both resource‐uptake traits and enemy release are thought to result in greater competitive ability and fitness of the successful invader compared to native species, ultimately leading to an increase in invader abundance at the expense of natives. Despite the popularity of these two hypotheses, the role of belowground enemy release in invasions has received interest only relatively recently, and often in the form of plant–soil feedback studies.

## The Plant–soil Feedback Model

Plant–soil feedback studies typically involve growing plants on soils in an initial “conditioning” phase, during which soil biota accumulate in a species‐specific manner in the plant rhizosphere. In a second “experimental” phase, plants are grown on soils conditioned by themselves (“home” soil), or by other species (“away” soil), or on their own soils which have or have not been sterilized. Differences in plant performance (typically measured as biomass) between home and away soils, or sterilized and unsterilized soils, are then interpreted as evidence for net negative (i.e., lower performance on home or unsterilized soil) or net positive (lower performance on away or sterilized soil) plant–soil feedback effects. Negative effects would indicate effects of soil pathogens on plant performance outweigh effects of any mutualists; positive effects would indicate the reverse. This approach has been used to assess the importance of (mainly pathogenic) soil biota in maintaining plant species coexistence (Petermann et al. 2008; Mangan et al. 2010), in driving species richness–productivity relationships (Maron et al. 2011; Schnitzer et al. 2011), and in explaining invasion when soil biota differ in the introduced range (van der Putten et al. 2007; Engelkes et al. 2008). For example, several studies have reported less negative or even positive effects of “home” soil biota on exotic/invasive species compared to native species (Klironomos 2002; Agrawal et al. 2005), and for invasives in invaded range soils compared to native range soils (Reinhart et al. 2003; Reinhart and Callaway 2004; Andonian et al. 2012; Maron et al. 2014). Moreover, there is some evidence that microbial communities associated with invasive alien plants differ from those of native species (Morriën and van der Putten 2013; Xiao et al. 2014). These results indicate that invasive alien plants may leave behind soilborne enemies (particularly microbial pathogens) in the introduced range, resulting in a fitness advantage compared to native species in invaded communities.

Crucially, however, these and many other plant–soil feedback studies do not consider how release from soilborne enemies can result in increased plant performance in the invaded range, and simply measure plant biomass as an estimate of plant performance. Plants may benefit directly from belowground enemy release through fitness increases resulting from reduced tissue damage and loss. However, I hypothesize that root traits that may be related to competition for resources and interference competition via allelopathy (the so‐called Novel Weapons hypothesis), could shift in response to enemy release, giving alien plants an indirect advantage. In the following paper, I outline how belowground enemy release could result in changes in alien plant root traits leading to invasion and discuss how putative changes in root traits of invasive plants due to enemy release could be assessed.

## Beyond Biomass: How Can Root Traits Respond to Belowground Enemy Release?

The degree of enemy release a species benefits from could depend at least partly on the species' traits, as postulated by the resource‐enemy release hypothesis: “High‐resource” traits reflecting adaptation to high‐resource environments should benefit most from enemy release in the alien range, as they invest less in defending tissues against natural enemies and more into growth (Blumenthal 2005, 2006). Conversely, the EICA hypothesis itself predicts that plant traits reflecting greater growth and competitive ability will evolve in the alien range, due to resource‐allocation shifts from redundant defense to growth. However, these interrelations between plant traits and enemy release have largely been considered aboveground. This is surprising, given the obvious fact that plant roots are vital for soil nutrient and water uptake, and thus affect plant growth and fitness (Bardgett et al. 2014). We might expect belowground plant enemies in the native range of species to exert strong impacts on the ability of plants to take up and compete for soil resources (de Kroon et al. 2012). Release from belowground enemies may therefore benefit alien plants through changes in root traits that result in greater soil resource uptake and therefore greater competitive ability compared to native species.

Just as a number of correlated leaf traits determine the light‐capturing and photosynthetic ability of plants (Wright et al. 2004), there is a suite of root traits that should determine soil resource uptake ability (Reich 2014). Plants with greater SRL—thinner/less dense roots with a greater length—should have a greater capacity to exploit resources (nutrients and water) from a greater volume of soil than plants with smaller SRL (Eissenstat 1992). Specific root length is correlated negatively with root diameter (Comas and Eissenstat 2009; McCormack et al. 2012), and positively with branching intensity (Comas and Eissenstat 2009) and root proliferation rates (Eissenstat 1991). Variation in these root traits is thus thought to represent a spectrum of resource uptake ability and plant growth (Reich 2014). However, plants with root traits reflecting a high‐resource‐uptake ability (highly branched, finer, less dense roots) will also likely face a cost of greater exposure to belowground enemies (Newsham et al. 1995; Eissenstat and Yanai 1997; Rasmann et al. 2011). The vulnerability of fine roots to soilborne enemies (bacteria, fungi, nematodes and other invertebrates) may inhibit root proliferation when plants are grown in monoculture with high soil‐enemy loads, thus reducing individual plant resource uptake and growth (“under‐rooting”; de Kroon et al. 2012). This belowground enemy effect has been put forward as an explanation for the phenomenon of “overyielding” in diversity experiments, whereby plants in species mixtures achieve greater biomass production than expected based on individual performance in monocultures, as they are less subject to the negative effects of their own soil biota in mixtures (Mommer et al. 2010; de Kroon et al. 2012; Hendriks et al. 2013).

**Figure 1:**
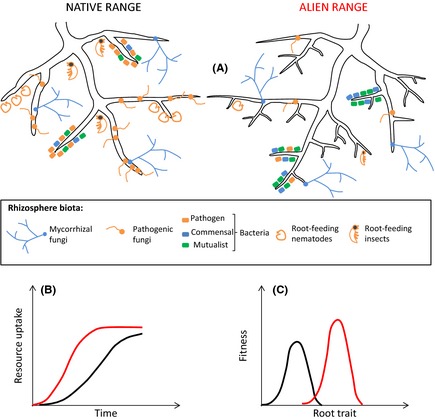
Schematic of the hypothesis that belowground enemy release in the rhizosphere of plants results in root‐trait changes, comparing the alien (red lines) and native range (red lines; A). Alien plants are expected to benefit from belowground enemy release, through expressing root traits such as greater branching and specific root length (A) which in turn allow greater rates of soil resource uptake in the alien compared to the native range (B). The shift in root traits toward values promoting greater resource uptake could be ecological or evolutionary, but should result in greater fitness for plants in the alien versus native range (C). The native range expectations in B and C could also represent native species in the alien range. While main groups of belowground enemies are depicted and are expected to be absent/less abundant in the alien range, root traits are expected to respond most strongly to release from fungal pathogens. Arbuscular mycorrhizal fungi should associate with plant roots at similar frequency in both ranges, but effects on plant growth may differ, with potential consequences for root‐trait expression (see “Integrating alternative belowground mechanisms explaining invasion success”).

If we consider plants in an alien range, they may have escaped belowground enemies (Fig. 1A) to the extent that there is no longer a cost to optimizing root traits and therefore resource uptake. Thus, we would expect root traits in the alien range to shift to values allowing greater resource uptake rates (e.g., greater branching, specific root length; Fig. 1A and B). This may then allow alien plants to perform better in monocultures, and give alien plants a competitive advantage in terms of resource‐acquisition ability, compared to native species that are still relatively “under‐rooted” due to enemy effects (Fig. 1C). To be clear, the hypothesis here is not that changes in root traits in response to belowground enemy release would result in altered placement and thus niche differentiation of plant roots. Instead, I hypothesize that belowground enemy release results in changes in root traits that increase an alien species' ability to pre‐empt and acquire soil resources over its neighbors, thus giving the alien a competitive advantage.

Despite the large number of plant–soil feedback studies conducted with invasive plants in recent years, none has considered how root traits may change in response to belowground enemy release, and how these changes relate to plant performance in the invaded range. The closest we have come so far is the comparison of basic biomass‐related traits, such as root mass and root:shoot ratio. Andonian et al. (2012) showed that *Centaurea solstitialis* had reduced root:shoot ratios in live compared to sterilized soils from the native European range, and from part of the alien range (Argentina), but not from other parts of the alien range (California and Chile), providing only partial evidence for shifts in biomass allocation due to differences in soil biota between ranges. Biogeographical studies specifically focusing on root morphological traits of native and alien range plant genotypes growing in native and alien range soils are absent.

**Figure 2:**
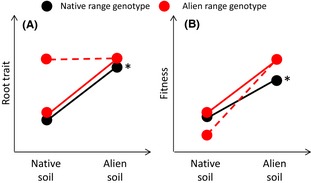
Shifts in root traits in response to belowground enemy release. In (A), the alien genotype should change its root traits to increase resource uptake in the alien range soil, compared to native range soil (red, solid line). Without evolution, native genotypes would be expected to respond in a similar manner (black). If enemy release resulted in evolution of root traits reflecting high‐resource uptake, then root traits of the alien genotype will not respond to the native range soil (red, dashed line). In (B) the fitness consequences are that, for ecological shifts in root traits, fitness should be higher for alien (and theoretically for native) genotypes in the alien range soil. If root traits evolved in response to soil‐enemy release, the root traits of alien genotypes should be better adapted to and therefore have higher fitness in alien range soils than in natives, but in the enemy‐bearing soil of the native range, alien genotypes would be maladapted (red dashed line) compared to native genotypes. Ethical considerations may prevent a fully reciprocal design, with native range genotypes in alien range soils omitted (*). However, disentangling ecological and evolutionary responses of root traits to belowground enemy release should still be feasible using the remaining genotype–soil combinations.

A shift in plant root traits to values that allow greater resource uptake could be evolutionary as well as ecological. The trade‐off described so far, between increasing resource uptake ability and increased physical exposure to enemy attack, could result in selection in favor of root traits allowing high‐resource uptake in the alien range (Fig. 2) Alternatively, a trade‐off between root traits related to growth rates, and root defense could result in selection in favor of genotypes with high‐resource‐uptake ability and against high defense genotypes in the alien range (effectively EICA belowground). The closest studies have got to testing evolution of root traits in the alien range under enemy release again involves measurement of root:shoot ratio (Kumschick et al. 2013), which is affected by both above‐ and belowground biomass, and is unlikely to reflect resource uptake ability clearly. Moreover, resistance to at least generalist herbivores has been found to be greater in the alien range for some species (Oduor et al. 2011; Kumschick et al. 2013), opposite to the prediction of the EICA hypothesis. In order to test for postintroduction evolution in root traits in response to belowground enemy release, a biogeographical approach will be necessary, ideally involving reciprocal growth experiments of alien and native range plant genotypes in native and alien range soils. Ethical considerations regarding introduction of new genotypes to the alien range may prevent a fully reciprocal design; however, evolution of root traits in the alien range genotypes should still be detectable by growing them on native and alien range soils (Fig. 2).

## Belowground Enemies: Who are the Potential Players?

There have been several thorough investigations into how the suite of belowground natural enemies of plants might differ between native and alien ranges, focusing on root‐feeding nematodes (Knevel et al. 2004; van der Putten et al. 2005), insect larvae (Briese 1989; Briese et al. 1994; Memmott et al. 2000), bacteria (Coats 2013), and pathogenic fungi (Reinhart et al. 2010; Callaway et al. 2011). Evidence for release of invasive species from nematodes is mixed. In the introduced ranges of Marram grass (*Ammophila arenaria*), fewer specialist nematodes have been found than in the native European range (Knevel et al. 2004; van der Putten et al. 2005). However, abundance of generalist root‐feeding nematodes rarely differed, and these were likely responsible for strong negative soil feedback effects on *A. arenaria* in South Africa. In Europe, Mairhofer et al. (2012) only found a lower abundance of root‐feeding nematodes in soils occupied by invasive compared to native plant species when accounting for plant biomass, with little relationship between nematode abundance and plant–soil feedback strength. Some alien plant species are released from specialized root‐feeding insect larvae. Purple viper's bugloss (*Echium plantagineum*) is an invasive plant attacked by the root crown weevil (*Mogulones larvatus*) in its native range, which has been recommended and released as a biocontrol agent (Sheppard et al. 2001; Buckley et al. 2005). Belowground insect herbivores are also few or absent on the invasive species *Cytisus scoparius* (Memmott et al. 2000), and *Onopordum* thistles in Australia (Briese 1989; Briese et al. 1994). Release from root herbivores that attack major roots (e.g., tap roots) will obviously have direct benefits to plants in the alien range. However, while plants in general are known to vary in direct and indirect root defense strategies (Rasmann et al. 2011), it is not known how root traits might alter in the absence of either specialist or generalist root‐feeding herbivores.

Soil bacteria and their interactions with plants are relatively understudied, although few bacteria are known to be plant pathogens (Raaijmakers et al. 2009). Molecular studies suggest that host plants can “culture” their own, specific rhizosphere bacterial communities (Marschner et al. 2001; Kowalchuk et al. 2002), and recent evidence points to differences in rhizosphere bacterial communities between native and invasive plants in the invaded range (Morriën and van der Putten 2013), and between plants in their native and alien ranges (Coats 2013). However, such differences should be interpreted with caution—a putative link to enemy release can only be identified if plant performance is negatively associated with abundance of a particular taxon in the native range, and if removal in the native range or absence of that taxon in the alien range results in increased plant performance. If rhizosphere bacterial communities show differences between alien and native ranges, but the bacteria involved are largely nonpathogenic, then such differences may be of little consequence to root traits related to resource uptake and alien plant performance.

Fungi are better studied as plant pathogens than are bacteria, and several studies have presented evidence that invasive plant species are likely to have become invasive due to release from specific soil fungal pathogens (Reinhart et al. 2003; Reinhart and Callaway 2004, 2006). For example, growth and survival of seedlings of the North American Black Cherry (*Prunus serotina*) are less negatively affected by soil biota in soils from underneath adults in the invaded European range, than in the native north American range (Reinhart et al. 2003). Subsequent work has shown that *Pythium* taxa in the invaded range have less virulent effects on *P. serotina* seedlings than taxa from the native range (Reinhart et al. 2010), indicating that release from virulent fungal pathogens' results has increased plant performance in the invaded range. In contrast, *Pythium* species from the native range of the leguminous tree *Robinia pseudoacacia* were not more virulent than taxa from the invaded ranges (Callaway et al. 2011); the negative soil feedback effects observed in the native range were likely due to other unidentified pathogens.

Despite evidence for soil fungal pathogen release, and for weaker plant–soil feedback effects in general for invasive plants (Kulmatiski et al. 2008), the response of root traits to fungal pathogen release has yet to be explored. As soil fungal pathogens infect plants *via* entry at the root surface, they are the group of belowground enemies whose effects are most likely to represent a cost to plants that increase root length and surface area to take up water and nutrients. Thus, we would expect plants to alter their root traits most in response to the absence of fungal pathogens in the invaded range. Efforts to test the hypotheses put forward here should therefore focus on identifying, isolating, and manipulating putative soil fungal pathogen species that are absent in the alien range of the plant species being studied. Moreover, there is some evidence from aboveground to suggest that plant species are less likely to share fungal pathogen release when they are less phylogenetically related (Gilbert and Webb 2007). If this is reflected belowground, then we would expect that release from soil fungal pathogens and subsequent root‐trait changes are more likely when the alien is more phylogenetically distant from natives.

## Integrating Alternative Belowground Mechanisms Explaining Invasion Success

I have focused on how belowground enemy release might result in changes in root traits that increase resource uptake ability, giving alien invasive plants a fitness advantage over competing native species. However, release from soilborne enemies and greater resource uptake ability are not the only potentially interlinked belowground mechanisms put forward to explain invasion success. Two other prominent hypotheses are the “Novel Weapons” hypothesis (Callaway and Ridenour 2004) and the “Enhanced Mutualism” hypothesis (Reinhart and Callaway 2006).

The Novel Weapons hypothesis postulates that chemical compounds produced by alien invasive plants that are unique in the invaded range may provide allelopathic, defense, or antimicrobial advantages over native competitors. These novel compounds can enter soils as litter from aboveground plant parts or *via* plant roots (from turnover of fine roots and/or as exudates). Effects of novel compounds on native competitors include decreased seed germination and plant growth (He et al. 2009; Zheng et al. 2015) and increased mortality (Inderjit et al., 2011), representing a form of interference competition. The weapons are “novel” in the alien range, because the native competitors are evolutionarily naïve to the compounds produced by the invader; more experienced competitors co‐occurring with the invader in its native range should be less negatively affected by the invader's weapons (Ridenour and Callaway 2004; He et al. 2009). Recently, Zheng et al. (2015) showed that the invasive plant *Chromolaena odorata* (in China) might have evolved increased production of allelopathic compounds with antimicrobial properties in aboveground plant material in response to altered enemy regimes postintroduction, leading to increased competitive ability against naïve native plants. Thus, enemy release and novel weapons might be interlinked drivers of invasion success.

**Figure 3:**
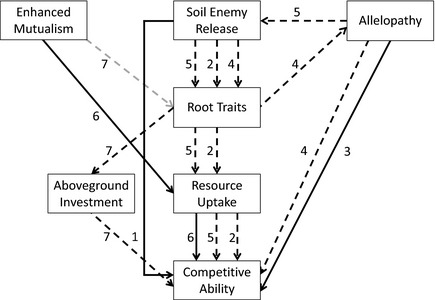
Schematic of how belowground enemy release and root traits may be interrelated with allelopathy or enhanced mutualism as mechanisms driving invasion success. Increased competitive ability as a result of enemy release could be direct (1), or indirect *via* root‐trait changes that increase soil resource uptake in the alien range (2). Increased competitive ability can result independently from allelopathy (3), or as a result of changes in root traits due to enemy release that result in increased concentrations of allelopathic compounds in the soil, and suppressive effects on natives in the alien range (4). If allelopathic compounds affect naïve pathogenic soil biota, then allelopathy will lead to effective enemy release in the alien range, and potentially shifts in root traits allowing greater resource uptake (5). Enhanced mutualism in the alien range could be of direct benefit through increased resource uptake (6), or indirect if alien plants invest less in costly, finer root growth; reinvestment into aboveground growth would then enhance competitive ability (7). All effects are positive (black), except the negative effect of mutualist enhancement on resource uptake‐related root traits (gray). Direct effects of belowground mechanisms on competitive ability are shown by solid lines; indirect effects are dashed lines.

While the recent study of Zheng et al. (2015) suggests a link between EICA and evolution of novel weapons in aboveground plant parts, the interrelatedness of belowground enemy release, belowground allelochemical production, and root traits in invasive plants has yet to be explored. For example, it is possible that in the alien range, belowground enemy release allows plants to produce finer, highly branched roots; such roots have a high turnover, and would release a greater quantity of exudate into rhizosphere soils per unit volume, than in the native range. If the species produced novel allelochemicals in the roots in the invaded range, then those plants could have a greater negative effect on native competitors than in the native range (Fig. 3). Thus, shifts in root traits in response to belowground enemy release could result in selection of genotypes with greater competitive ability, both through increased allelopathy and through greater soil resource uptake. When allelopathic chemicals have antimicrobial properties, it is possible that the relationship between enemy release, root traits, and allelopathy is altered, such that naïve soil biota in the alien range with potential pathogenic effects are reduced in abundance in rhizosphere soils of the invader. This could then lead to effective enemy release, and proliferation of fine, highly branched roots that increase resource uptake (Fig. 3).

Instead of belowground enemies driving invasive plant success, the enhanced mutualism hypothesis posits that alien plants may benefit through greater positive effects of mutualists present in the alien range on plant performance than in the native range (Reinhart and Callaway 2006). Even though mutualisms between mycorrhizal fungi and plants are near ubiquitous, the strength and directions of interactions between plant and fungus are known to vary according to species identity of either organism (e.g., for arbuscular mycorrhizal fungi, AMF‐ Klironomos 2003), and so new combinations of plant and soil symbiont could increase alien plant fitness and trigger invasion. There is some evidence that *Robinia pseudoacacia* recruits a wide range of N‐fixing bacterial symbionts across its alien range, but benefits from more positive interactions with AMF from its native range than from the alien range (Callaway et al. 2011). Recent work from New Zealand has shown that invasive pinaceae tree species associate with novel alien ectomycorrhizal species (Wood et al. 2015), but a link to enhanced plant performance has yet to be made. A potential consequence of enhanced mutualism for root traits would be a shift in the opposite direction to enemy release. Enhanced mutualism could reduce the need for the plant to invest in roots that increase resource uptake ability, leading to less branching and coarser roots. As with the effects of natural enemies, changes in root traits in response to enhanced mutualism have yet to be explored, but it is plausible that a reduction in finer roots with high construction costs may allow alien plants to allocate more resources to aboveground growth (Fig. 3).

## Approaches: Measuring Root Traits and Disentangling Belowground Drivers of Plant Invasions

Despite the large number of studies testing whether plant–soil feedbacks are related to invasion success, no study has sought to understand how belowground enemy release can mechanistically result in alien plants having a fitness advantage over natives, through changes in root traits and soil resource uptake. An important reason for this is the difficulty in measuring root traits and root architecture. To test the hypothesis that enemy release leads to shifts in root traits in the alien range, the simplest traits to measure are those based on root length (i.e., specific root length, root length density [expressed as length per unit volume of soil]), root diameter, and degree of branching (the number of forks, or the number of root tips). These root traits can be measured on plants growing alone by washing roots, scanning them, and analyzing the resulting images (e.g., using WinRHIZO^™^; Regent Instruments Inc., Canada). However, such work is time‐consuming, leads to some loss of finer roots, and is not well suited to plants growing in humus‐rich soils (due to entanglement and proliferation of roots in organic material), or to older and larger plants with more extensive root systems. The biggest disadvantage to measuring root traits directly *via* root washing is that it is often impossible to fully separate intact root systems of plants growing together in competition.

X‐ray micro‐computed tomography (*μ*CT) and automated processing of images using specialist software (e.g., RooTrak; Mairhofer et al. 2012, 2013) can allow visualization of the three‐dimensional architecture of plants growing in soil. More recently, *μ*CT has been tested as a method for measuring the root architecture of interacting plants in a study by Paya et al. (2015). While estimates of root surface area and the number of root tips from destructive harvesting (root washing and 2D scanning) and from constructed 3D *μ*CT images were strongly correlated, only 62% of root tips and 76% of total root surface area captured by the 3D images (Paya et al.^2015^). The study was able to detect differences in 3D spacing of roots between no‐competition and competition treatments. Paya et al. (2015) used artificial substrate and not real soil, due to problems of soil water retention and the consequent inability of *μ*CT scanning to differentiate between water in roots and water in the soil. In general, the rooting volume that can be scanned successfully using *μ*CT is still relatively small, and image acquisition and processing is sensitive to variations in substrate and root density, requiring calibration for different soils and plant species (Mairhofer et al. 2013; Paya et al. 2015). Nonetheless, the *μ*CT approach appears to be a promising method for assessing how root traits involving invasive plants under competition might differ between alien and native ranges as a result of enemy release.

The importance of changes in root traits in response to belowground enemy release would also need to be put into the context of other belowground hypotheses explaining invasions. Understanding whether root traits change in relation to belowground enemy release, enhanced mutualisms, or both would require careful separation of symbiont and enemy fractions of soil biotas, reinoculation of plants in the alien and native range, and assessment of plant performance and root traits. Separating mutualists from pathogen components can be achieved most easily for arbuscular mycorrhizal fungal spores using fine‐mesh sieves (AMF spores are >20 *μ*m; pathogenic fungi and bacteria are usually <20 *μ*m; Wagg et al. 2014), and a decreasing mesh‐size approach has been used to separate such components in previous plant–soil feedback studies (Klironomos 2002; Callaway et al. 2011).

To test for allelopathy, experiments typically involve applying a leachate fluid from organic material of the target species to plants of potential competitor species, or growing plants under competition with and without activated carbon, which absorbs labile allelopathic chemicals released into the soil, thus minimizing allelopathic effects. These approaches could be used in conjunction with measuring root traits in native and alien range soils that have or have not been sterilized, to tease apart the roles of allelopathy, belowground enemy release, and root‐trait changes in explaining increased competitive ability. However, there are potential undesired side effects of activated carbon on soil physicochemical properties and on soil microbial communities (Weisshuhn and Prati 2009; Nolan et al. 2015). Producing realistic concentrations of allelopathic compounds in leachates can also be difficult. However, to test whether allelopathy and root‐trait changes are interrelated drivers of invasion, one could assess whether variation in root traits relates to per unit volume concentrations of potential allelopathic chemicals in soils: A positive relationship would indicate that plants with finer, more branched roots release more compounds into the surrounding soil volume.

It will be challenging to test whether belowground enemy release results in root‐trait changes and then increased allelopathy, or whether allelopathy affects naïve soil pathogens which then affect root traits. One approach could involve using next‐generation sequencing to identify microbial taxa in alien range soils that show a marked decrease in abundance in the rhizosphere of the invader, and to identify similar decreases in soils treated only with root extracts. Soils containing invader plants could be inoculated with root extracts, root traits could be measured, and comparisons made to plants receiving the same treatment in the native range. We would then expect extract‐altered soil microbial communities to lead to root trait changes in the alien, but not in the native range if changes are due to soil biota that are naïve to the invader's allelochemicals.

Ultimately, to show that belowground enemy release leads to shifts in root traits that give alien plants a fitness advantage, one would need to link changes in root traits to competition for nutrients and plant performance. Analyzing nutrient uptake will give a more direct measure of how root‐trait changes affect plant–plant competition for nutrients than simply measuring biomass as a proxy of competitive ability, and would ideally be measured repeatedly throughout the plants' life cycles to capture the dynamics of resource acquisition as alien and native plants grow, compete, and reproduce (Trinder et al. 2013). To assess whether root‐trait changes result in greater nutrient uptake by alien plants than by competing natives, one could use isotopically labeled nutrient fertilizers, such as ^15^N‐labeled ammonium (NH_4_^+^), nitrate (NO_3_^−^), both combined (NH_4_NO_3_), or ^15^N‐labeled amino acids. One can then analyse the percentage ^15^N content in plant material to quantify nitrogen uptake, and compare uptake of invasive alien and native plants growing under competition. Differences in N uptake could then be regressed against differences in root traits; one would expect greater differences in root traits between alien invasive and native plants to be reflected in greater N uptake by the alien compared to the native. In addition, comparisons of invasive plant N uptake could be made between alien and native ranges. If root‐trait changes due to enemy release occur in the alien range, then one would also expect relatively higher nutrient uptake by invasive plants in the alien than the native range.

## Conclusions

Our knowledge of interrelated causes of plant invasions involving traits and biotic interactions has a clear aboveground bias; the “hidden half” of plants belowground is still very much hidden in terms of how alien plants interact with and change in response to novel biota in the alien range. To address this bias, I have outlined a novel hypothesis of how belowground enemy release can result in increased plant competitive ability and performance in the alien range, via shifts in root traits that increase resource uptake ability, and allow alien plants to outcompete native species. Such shifts in root traits could be evolutionary as well as ecological, but in either case, efforts to understand how root traits change will be most successful when a biogeographic approach is taken, ideally using plant genotypes and soils from both the alien and native range. I also propose that invasive alien plants are most likely to benefit *via* root traits from fungal pathogen release than from other belowground enemies, because root attack by fungal pathogens should be most directly related to root architecture and morphology. Moreover, the potential for escaping species‐specific soil fungal pathogens is likely to be high.

Carefully designed experiments will be required to disentangle the role of belowground enemy release and resulting changes in root traits from other potential belowground drivers of invasion, such as allelopathy and enhanced mutualism. However, if allelopathic compounds are produced as root exudates and from fine‐root decomposition, it is plausible that allelopathy, enemy release, and root‐trait changes represent interdependent (and not competing) drivers of invasion. Next‐generation sequencing could be employed in combination with plant growth experiments to understand how soil biota respond to changes in root traits, and to potentially allelopathic root extracts. Such approaches could also be used to identify which fungal pathogen taxa are present and interacting with invasive plants in the alien range (Day et al. 2015) and in the native range, and whether native range pathogens have a higher virulence. Measuring root traits to test the hypothesis put forward can be difficult, particularly when plants are growing under competition; however, advances in *μ*‐CT scanning and image processing methods should make it feasible to capture root‐trait values for plants growing in competition in situ in soil. These methods, combined with linking root traits to measurements of nutrient uptake using isotope‐labeling, should shed light on whether belowground enemy release drives alien plant invasion success *via* changes in root traits linked to soil resource uptake.

## Conflict of Interest

None declared.

## References

[ece31725-bib-0001] Agrawal, A. A. , P. M. Kotanen , C. E. Mitchell , A. G. Power , W. Godsoe , and J. Klironomos . 2005 Enemy release? An experiment with congeneric plant pairs and diverse above‐ and belowground enemies. Ecology 86:2979–2989. doi:10.1890/05‐0219.

[ece31725-bib-0002] Andonian, K. , J. L. Hierro , L. Khetsuriani , P. I. Becerra , G. Janoyan , D. Villareal , et al. 2012 Geographic mosaics of plant‐soil microbe interactions in a global plant invasion. J. Biogeogr. 39:600–608. doi:10.1111/j.1365‐2699.2011.02629.x.

[ece31725-bib-0003] Baker, H. 1965 Characteristics and modes of origin of weeds Pp. 147–172 *in* Baker H. G., Stebbins G. L., eds. The genetics of colonizing species. Academic Press, New York.

[ece31725-bib-0004] Bardgett, R. D. , L. Mommer , and F. T. De Vries . 2014 Going underground: root traits as drivers of ecosystem processes. Trends Ecol. Evol. 29:692–699. doi:10.1016/j.tree.2014.10.006. 25459399 10.1016/j.tree.2014.10.006

[ece31725-bib-0005] Blossey, B. , and R. Notzold . 1995 Evolution of increased competitive ability in invasive nonindigenous plants‐ a hypothesis. J. Ecol. 83:887–889. doi:10.2307/2261425.

[ece31725-bib-0006] Blumenthal, D. 2005 Interrelated causes of plant invasion. Science 310:243–244. doi:10.1126/science.1114851. 16224008 10.1126/science.1114851

[ece31725-bib-0007] Blumenthal, D. M. 2006 Interactions between resource availability and enemy release in plant invasion. Ecol. Lett. 9:887–895. doi:10.1111/j.1461‐0248.2006.00934.x. 16796578 10.1111/j.1461-0248.2006.00934.x

[ece31725-bib-0008] Briese, D. T. 1989 Natural enemies of carduine thistles in New South Wales. J. Aust. Entomol. Soc. 28:125–134. doi:10.1111/j.1440‐6055.1989.tb01209.x.

[ece31725-bib-0009] Briese, D. T. , A. W. Sheppard , H. Zwolfer , and P. E. Boldt . 1994 Structure of the phytophagous insect fauna of *Onopordum* thistles in the northern Mediterranean Basin. Biol. J. Linn. Soc. 53:231–253. doi:10.1111/j.1095‐8312.1994.tb01011.x.

[ece31725-bib-0010] Buckley, Y. M. , M. Rees , A. W. Sheppard , and M. J. Smyth . 2005 Stable coexistence of an invasive plant and biocontrol agent: a parameterized coupled plant‐herbivore model. J. Appl. Ecol. 42:70–79. doi:10.1111/j.1365‐2664.2005.00991.x.

[ece31725-bib-0011] Callaway, R. M. , and W. M. Ridenour . 2004 Novel weapons: invasive success and the evolution of increased competitive ability. Front. Ecol. Environ. 2:436–443. doi:10.1890/1540‐9295(2004)002[0436:NWISAT]2.0.CO;2.

[ece31725-bib-0012] Callaway, R. M. , E. J. Bedmar , K. O. Reinhart , C. Gomez Silvan , and J. Klironomos . 2011 Effects of soil biota from different ranges on *Robinia* invasion: acquiring mutualists and escaping pathogens. Ecology 92:1027–1035. doi:10.1890/10‐0089.1. 21661564 10.1890/10-0089.1

[ece31725-bib-0013] Coats, V. C. 2013 Microbial associates of Berberis thunbergii (Japanese Barberry). Ph.D. dissertation, University of Maine, Ann Arbor: ProQuest/UMI.

[ece31725-bib-0014] Comas, L. H. , and D. M. Eissenstat . 2009 Patterns in root trait variation among 25 co‐existing North American forest species. New Phytol. 182:919–928. doi:10.1111/j.1469‐8137.2009.02799.x. 19383105 10.1111/j.1469-8137.2009.02799.x

[ece31725-bib-0150] Davidson, A. M. , M. Jennions , and A. B. Nicotra . 2011 Do invasive species show higher phenotypic plasticity than native species and if so, is it adaptive? A meta‐analysis. Ecol. Lett. 14:419–431. doi:10.1111/j.1461‐0248.2011.01596.x. 21314880 10.1111/j.1461-0248.2011.01596.x

[ece31725-bib-0015] Dawson, W. , M. Fischer , and M. van Kleunen . 2011 The maximum relative growth rate of common UK plant species is positively associated with their global invasiveness. Glob. Ecol. Biogeogr. 20:299–306. doi:10.1111/j.1466‐8238.2010.00599.x.

[ece31725-bib-0016] Dawson, W. , M. Fischer , and M. van Kleunen . 2012a Common and rare plant species respond differently to fertilisation and competition, whether they are alien or native. Ecol. Lett. 15:873–880. doi:10.1111/j.1461‐0248.2012.01811.x. 22676338 10.1111/j.1461-0248.2012.01811.x

[ece31725-bib-0017] Dawson, W. , R. P. Rohr , M. van Kleunen , and M. Fischer . 2012b Alien plant species with a wider global distribution are better able to capitalize on increased resource availability. New Phytol. 194:859–867. doi:10.1111/j.1469‐8137.2012.04104.x. 22409575 10.1111/j.1469-8137.2012.04104.x

[ece31725-bib-0018] Day, N. J. , K. E. Dunfield , and P. M. Antunes . 2015 Temporal dynamics of plant‐soil feedback and root‐associated fungal communities over 100 years of invasion by a non‐native plant. J. Ecol.. doi:10.1111/1365‐2745.12459.

[ece31725-bib-0019] Eissenstat, D. M. 1991 On the relationship between specific root length and the rate of root proliferation‐ a field‐study using *Citrus* rootstocks. New Phytol. 118:63–68.

[ece31725-bib-0020] Eissenstat, D. M. 1992 Costs and benefits of constructing roots of small diameter. J. Plant Nutr. 15:763–782. doi:10.1080/01904169209364361.

[ece31725-bib-0021] Eissenstat, D. M. , and R. D. Yanai 1997 The ecology of root lifespan Pp. 1–60 *in* Begon M., Fitter A. H., eds. Advances in ecological research, vol. 27. Academic Press, New York

[ece31725-bib-0022] Elton, C. 1958 The ecology of invasions by animals and plants. Methuen, London.

[ece31725-bib-0023] Engelkes, T. , E. Morrien , K. J. F. Verhoeven , T. M. Bezemer , A. Biere , J. A. Harvey , et al. 2008 Successful range‐expanding plants experience less above‐ground and below‐ground enemy impact. Nature 456:946–948. doi:10.1038/nature07474. 19020504 10.1038/nature07474

[ece31725-bib-0024] Gilbert, G. S. , and C. O. Webb . 2007 Phylogenetic signal in plant pathogen‐host range. Proc. Natl Acad. Sci. USA 104:4979–4983. doi:10.1073/pnas.0607968104. 17360396 10.1073/pnas.0607968104 PMC1829250

[ece31725-bib-0025] Grotkopp, E. , and M. Rejmanek . 2007 High seedling relative growth rate and specific leaf area are traits of invasive species: phylogenetically independent contrasts of woody angiosperms. Am. J. Bot. 94:526–532. doi:10.3732/ajb.94.4.526. 21636422 10.3732/ajb.94.4.526

[ece31725-bib-0026] Grotkopp, E. , M. Rejmanek , and T. L. Rost . 2002 Toward a causal explanation of plant invasiveness: seedling growth and life‐history strategies of 29 pine (*Pinus*) species. Am. Nat. 159:396–419. doi:10.1086/338995. 18707424 10.1086/338995

[ece31725-bib-0027] He, W.‐M. , Y.‐L. Feng , W. M. Ridenour , G. C. Thelen , J. L. Pollock , A. Diaconu , et al. 2009 Novel weapons and invasion: biogeographic differences in the competitive effects of *Centaurea maculosa* and its root exudate (±) catechin. Oecologia 159:803–815. doi:10.1007/s00442‐008‐1234‐4. 19219462 10.1007/s00442-008-1234-4

[ece31725-bib-0028] Hendriks, M. , L. Mommer , H. de Caluwe , A. E. Smit‐Tiekstra , W. H. van der Putten , and H. de Kroon . 2013 Independent variations of plant and soil mixtures reveal soil feedback effects on plant community overyielding. J. Ecol. 101:287–297. doi:10.1111/1365‐2745.12032.

[ece31725-bib-0201] Inderjit, H. , C. Evans , D. Crocoll , R. Bajpai , Y.‐L. Kaur , C. Feng , J. T. Silva , A. Carreon , J. Gershenzon Valiente‐Banuet , and R. M. Callaway . 2011 Volatile chamicals from leaf litter are associated with invasiveness of a Neotropical weed in Asia. Ecology 92:316–324. doi:10.1890/10‐0400.1. 21618911 10.1890/10-0400.1

[ece31725-bib-0029] Keane, R. M. , and M. J. Crawley . 2002 Exotic plant invasions and the enemy release hypothesis. Trends Ecol. Evol. 17:164–170. doi:10.1016/S0169‐5347(02)02499‐0.

[ece31725-bib-0030] van Kleunen, M. , W. Dawson , D. Schlaepfer , J. M. Jeschke , and M. Fischer . 2010a Are invaders different? A conceptual framework of comparative approaches for assessing determinants of invasiveness. Ecol. Lett. 13:947–958. doi:10.1111/j.1461‐0248.2010.01503.x. 20576028 10.1111/j.1461-0248.2010.01503.x

[ece31725-bib-0031] van Kleunen, M. , E. Weber , and M. Fischer . 2010b A meta‐analysis of trait differences between invasive and non‐invasive plant species. Ecol. Lett. 13:235–245. doi:10.1111/j.1461‐0248.2009.01418.x. 20002494 10.1111/j.1461-0248.2009.01418.x

[ece31725-bib-0032] Klironomos, J. N. 2002 Feedback with soil biota contributes to plant rarity and invasiveness in communities. Nature 417:67–70. doi:10.1038/417067a. 11986666 10.1038/417067a

[ece31725-bib-0033] Klironomos, J. N. 2003 Variation in plant response to native and exotic arbuscular mycorrhizal fungi. Ecology 84:2292–2301. doi:10.1890/02‐0413.

[ece31725-bib-0034] Knevel, I. C. , T. Lans , F. B. J. Menting , U. M. Hertling , and W. H. van der Putten . 2004 Release from native root herbivores and biotic resistance by soil pathogens in a new habitat both affect the alien Ammophila arenaria in South Africa. Oecologia 141:502–510. doi:10.1007/s00442‐004‐1662‐8. 15309610 10.1007/s00442-004-1662-8

[ece31725-bib-0035] Kowalchuk, G. A. , D. S. Buma , W. de Boer , P. G. L. Klinkhamer , and J. A. van Veen . 2002 Effects of aboveground plant species composition and diversity on the diversity of soil‐borne microorganisms. Antonie Van Leeuwenhoek 81:509–520. doi:10.1023/A:1020565523615. 12448746 10.1023/a:1020565523615

[ece31725-bib-0036] de Kroon, H. , M. Hendriks , J. van Ruijven , J. Ravenek , F. M. Padilla , E. Jongejans , et al. 2012 Root responses to nutrients and soil biota: drivers of species coexistence and ecosystem productivity. J. Ecol. 100:6–15. doi: 10.1111/j.1365‐2745.2011.01906.x

[ece31725-bib-0037] Kulmatiski, A. , K. H. Beard , J. R. Stevens , and S. M. Cobbold . 2008 Plant‐soil feedbacks: a meta‐analytical review. Ecol. Lett. 11:980–992. doi:10.1111/j.1461‐0248.2008.01209.x. 18522641 10.1111/j.1461-0248.2008.01209.x

[ece31725-bib-0038] Kumschick, S. , R. A. Hufbauer , C. Alba , and D. M. Blumenthal . 2013 Evolution of fast‐growing and more resistant phenotypes in introduced common mullein (*Verbascum thapsus*). J. Ecol. 101:378–387. doi:10.1111/1365‐2745.12044.

[ece31725-bib-0039] Mairhofer, S. , S. Zappala , S. R. Tracy , C. Sturrock , M. Bennett , S. J. Mooney , et al. 2012 RooTrak: automated recovery of three‐dimensional plant root architecture in soil from X‐ray microcomputed tomography images using visual tracking. Plant Physiol. 158:561–569. doi: 10.1104/pp.111.186221. 22190339 10.1104/pp.111.186221 PMC3271750

[ece31725-bib-0040] Mairhofer, S. , S. Zappala , S. R. Tracy , C. Sturrock , M. Bennett , S. J. Mooney , et al. 2013 Recovering complete plant root system architectures from soil via X‐ray *μ*‐computed tomography. Plant Methods 9:8. doi:10.1186/1746‐4811‐9‐8. 23514198 10.1186/1746-4811-9-8 PMC3615952

[ece31725-bib-0041] Mangan, S. A. , S. A. Schnitzer , E. A. Herre , K. M. L. Mack , M. C. Valencia , E. I. Sanchez , et al. 2010 Negative plant‐soil feedback predicts tree‐species relative abundance in a tropical forest. Nature 466:752–755. doi:10.1038/nature09273. 20581819 10.1038/nature09273

[ece31725-bib-0042] Maron, J. L. , M. Marler , J. N. M Klironomos , and C. C. Cleveland . 2011 Soil fungal pathogens and the relationship between plant diversity and productivity. Ecol. Lett. 14:36–41. doi: 10.1111/j.1461‐0248.2010.01547.x. 21073641 10.1111/j.1461-0248.2010.01547.x

[ece31725-bib-0043] Maron, J. L. , J. Klironomos , L. Waller , and R. M. Callaway . 2014 Invasive plants escape from suppressive soil biota at regional scales. J. Ecol. 102:19–27. doi:10.1111/1365‐2745.12172.

[ece31725-bib-0044] Marschner, P. , C.‐H. Yang , R. Lieberei , and D. E. Crowley . 2001 Soil and plant specific effects on bacterial community composition in the rhizosphere. Soil Biol. Biochem. 33:1437–1445. doi:10.1016/S0038‐0717(01)00052‐9.

[ece31725-bib-0045] McCormack, M. L. , T. S. Adams , E. A. H. Smithwick , and D. M. Eissenstat . 2012 Predicting fine root lifespan from plant functional traits in temperate trees. New Phytol. 195:823–831. doi:10.1111/j.1469‐8137.2012.04198.x. 22686426 10.1111/j.1469-8137.2012.04198.x

[ece31725-bib-0046] Memmott, J. , S. V. Fowler , Q. Paynter , A. W. Sheppard , and P. Syrett . 2000 The invertebrate fauna on broom, *Cytisus scoparius*, in two native and two exotic habitats. Acta Oecol. Int. J. Ecol. 21:213–222. doi:10.1016/S1146‐609X(00)00124‐7.

[ece31725-bib-0047] Mommer, L. , J. van Ruijven , H. de Caluwe , A. E. Smit‐Tiekstra , C. A. M. Wagemaker , N. J. Ouborg , et al. 2010 Unveiling below‐ground species abundance in a biodiversity experiment: a test of vertical niche differentiation among grassland species. J. Ecol. 98:1117–1127. doi:10.1111/j.1365‐2745.2010.01702.x.

[ece31725-bib-0048] Morriën, E. , and W. H. van der Putten . 2013 Soil microbial community structure of range‐expanding plant species differs from co‐occurring natives. J. Ecol. 101:1093–1102. doi:10.1111/1365‐2745.12117.

[ece31725-bib-0049] Newsham, K. K. , A. H. Fitter , and A. R. Watkinson . 1995 Multi‐functionality and biodiversity in arbuscular mycorrhizas. Trends Ecol. Evol. 10:407–411. doi:10.1016/S0169‐5347(00)89157‐0. 21237085 10.1016/s0169-5347(00)89157-0

[ece31725-bib-0050] Nolan, N. E. , A. Kulmatiski , K. H. Beard , and J. M. Norton . 2015 Activated carbon decreases invasive plant growth by mediating plant‐microbe interactions. AoB Plants 7:plu072. doi: 10.1093/aobpla/plu072. 25387751 10.1093/aobpla/plu072 PMC4303759

[ece31725-bib-0051] Oduor, A. M. O. , R. A. Lankau , S. Y. Strauss , and J. M. Gomez . 2011 Introduced *Brassica nigra* populations exhibit greater growth and herbivore resistance but less tolerance than native populations in the native range. New Phytol. 191:536–544. doi:10.1111/j.1469‐8137.2011.03685. 21410474 10.1111/j.1469-8137.2011.03685.x

[ece31725-bib-0202] Palacio‐Lopez, K. , and E. Gianoli . 2011 Invasive plants do not display greater phenotypic plasticity than their native or non‐invasive counterparts: a meta‐analysis. Oikos 120:1393–1401. doi:10.1111/j.1600‐0706.2010.19114.x.

[ece31725-bib-0052] Parepa, M. , M. Fischer , and O. Bossdorf . 2013 Environmental variability promotes plant invasion. Nat. Commun. 4:1604. doi:10.1038/ncomms2632. 23511469 10.1038/ncomms2632

[ece31725-bib-0053] Paya, A. M. , J. L. Silverberg , J. Padgett , and T. L. Bauerle . 2015 X‐ray computed tomography uncovers root‐root interactions: quantifying spatial relationships between interacting root systems in three dimensions. Front. Plant Sci. 6:274. doi:10.3389/fpls.2015.00274. 25972880 10.3389/fpls.2015.00274 PMC4413727

[ece31725-bib-0054] Petermann, J. S. , A. J. F. Fergus , L. A. Turnbull , and B. Schmid . 2008 Janzen‐Connell effects are widespread and strong enough to maintain diversity in grasslands. Ecology 89:2399–2406. doi:10.1890/07‐2056.1. 18831160 10.1890/07-2056.1

[ece31725-bib-0055] van der Putten, W. H. , G. W. Yeates , H. Duyts , C. S. Reis , and G. Karssen . 2005 Invasive plants and their escape from root herbivory: a worldwide comparison of the root‐feeding nematode communities of the dune grass *Ammophila arenaria* in natural and introduced ranges. Biol. Invasions 7:733–746. doi:10.1007/s10530‐004‐1196‐3.

[ece31725-bib-0056] van der Putten, W. H. , J. N. Klironomos , and D. A. Wardle . 2007 Microbial ecology of biological invasions. ISME J. 1:28–37. doi:10.1038/ismej.2007.9. 18043611 10.1038/ismej.2007.9

[ece31725-bib-0057] Pyšek, P. , and D. M. Richardson . 2007 Traits associated with invasiveness in alien plants: where do we stand? Pp. 97–125 *in* Nentwig W., ed. Biological invasions. Springer, New York.

[ece31725-bib-0058] Raaijmakers, J. M. , T. C. Paulitz , C. Steinberg , C. Alabouvette , and Y. Moenne‐Loccoz . 2009 The rhizosphere: a playground and battlefield for soilborne pathogens and beneficial microorganisms. Plant Soil 321:341361.

[ece31725-bib-0059] Rasmann, S. , T. L. Bauerle , K. Poveda , and R. Vannette . 2011 Predicting root defence against herbivores during succession. Funct. Ecol. 25:368–379. doi: 10.1111/j.1365‐2435.2010.01811.x.

[ece31725-bib-0060] Reich, P. B. 2014 The world‐wide ‘fast‐slow’ plant economics spectrum: a traits manifesto. J. Ecol. 102:275–301. doi:10.1111/1365‐2745.12211.

[ece31725-bib-0061] Reinhart, K. O. , and R. M. Callaway . 2004 Soil biota facilitate exotic *Acer* invasions in Europe and North America. Ecol. Appl. 14:1737–1745. doi:10.1890/03‐5204.

[ece31725-bib-0062] Reinhart, K. O. , and R. M. Callaway . 2006 Soil biota and invasive plants. New Phytol. 170:445–457. doi:10.1111/j.1469‐8137.2006.01715.x. 16626467 10.1111/j.1469-8137.2006.01715.x

[ece31725-bib-0063] Reinhart, K. O. , A. Packer , W. H. Van der Putten , and K. Clay . 2003 Plant‐soil biota interactions and spatial distribution of black cherry in its native and invasive ranges. Ecol. Lett. 6:1046–1050. doi:10.1046/j.1461‐0248.2003.00539.

[ece31725-bib-0064] Reinhart, K. O. , T. Tytgat , W. H. van der Putten , and K. Clay . 2010 Virulence of soil‐borne pathogens and invasion by *Prunus serotina* . New Phytol. 186:484–495. doi:10.1111/j.1469‐8137.2009.03159.x. 20100208 10.1111/j.1469-8137.2009.03159.x

[ece31725-bib-0065] Rejmanek, M. , and D. M. Richardson . 1996 What attributes make some plant species more invasive? Ecology 77:1655–1661. doi:10.2307/2265768.

[ece31725-bib-0066] Schnitzer, S. A. , J. N. Klironomos , J. HilleRisLambers , L. L. Kinkel , P. B. Reich , K. Xiao , et al. 2011 Soil microbes drive the classic plant diversity‐productivity pattern. Ecology 92:296–303. doi:10.1890/10‐0773.1. 21618909 10.1890/10-0773.1

[ece31725-bib-0067] Seabloom, E. W. , E. T. Borer , Y. M. Buckley , E. E. Cleland , K. F. Davies , J. Firn , et al. 2015 Plant species’ origin predicts dominance and response to nutrient enrichment and herbivores in global grasslands. Nat. Commun. 6:7710. doi:10.1038/ncomms8710. 26173623 10.1038/ncomms8710 PMC4518311

[ece31725-bib-0068] Sheppard, A. W. , M. J. Smyth , and A. Swirepik . 2001 The impact of a root‐crown weevil and pasture competition on the winter annual *Echium plantagineum* . J. Appl. Ecol. 38:291–300. doi:10.1046/j.1365‐2664.2001.00583.x.

[ece31725-bib-0069] Trinder, C. J. , R. W. Brooker , and D. Robinson . 2013 Plant ecology's guilty little secret: understanding the dynamics of competition. Funct. Ecol. 27:918–929. doi:10.1111/1365‐2435.12078.

[ece31725-bib-0070] Wagg, C. , S. F. Bender , F. Widmer , and M. A. van der Heijden . 2014 Soil biodiversity and soil community composition determine ecosystem multifunctionality. Proc. Natl Acad. Sci. USA 111:5266–5270. doi:10.1073/pnas.1320054111. 24639507 10.1073/pnas.1320054111 PMC3986181

[ece31725-bib-0071] Weisshuhn, K. , and D. Prati . 2009 Activated carbon may have undesired side effects for testing allelopathy in invasive plants. Basic Appl. Ecol. 10:500–507. doi:10.1016/j.baae.2008.10.009.

[ece31725-bib-0072] Wood, J. R. , I. A. Dickie , H. V. Moeller , D. A. Peltzer , K. I. Bonner , G. Rattray , et al. 2015 Novel interactions between non‐native mammals and fungi facilitate establishment of invasive pines. J. Ecol. 103:121–129. doi:10.1111/1365‐2745.12345.

[ece31725-bib-0073] Wright, I. J. , P. B. Reich , M. Westoby , D. D. Ackerly , Z. Baruch , F. Bongers , et al. 2004 The worldwide leaf economics spectrum. Nature 428:821–827. doi:10.1038/nature02403. 15103368 10.1038/nature02403

[ece31725-bib-0074] Xiao, H. F. , Y. L. Feng , D. A. Schaefer , and X. D. Yang . 2014 Soil fungi rather than bacteria were modified by invasive plants, and that benefited invasive plant growth. Plant Soil 378:253–264. doi:10.1007/s11104‐014‐2040‐x.

[ece31725-bib-0075] Zheng, Y.‐L. , Y.‐L. Feng , L.‐K. Zhange , R. M. Callaway , A. V. Banuet , D.‐Q. Luo , et al. 2015 Integrating novel chemical weapons and evolutionarily increased competitive ability in success of a tropical invader. New Phytol. 205:1350–1359. doi:10.1111/nph.13135. 25367824 10.1111/nph.13135

